# ﻿*Mazusdanxiacola* (Mazaceae), a distinct new species endemic to Danxia landform in Jiangxi Province, eastern China

**DOI:** 10.3897/phytokeys.199.85717

**Published:** 2022-06-03

**Authors:** Bo Li, Xin-Gui Le, Dao-Zhang Min, Lin Xu, Bin Chen

**Affiliations:** 1 Research Centre of Ecological Sciences, College of Agronomy, Jiangxi Agricultural University, Nanchang 330045, Jiangxi, China Jiangxi Agricultural University Nanchang China; 2 State Key Laboratory of Desert and Oasis Ecology, Xinjiang Institute of Ecology and Geography, Chinese Academy of Sciences, Urumqi 830011, Xinjiang, China Xinjiang Institute of Ecology and Geography, Chinese Academy of Sciences Urumqi China; 3 Yangjifeng National Nature Reserve Administration of Jiangxi Province, Guixi 335400, Jiangxi, China Yangjifeng National Nature Reserve Administration of Jiangxi Province Guixi China; 4 Eastern China Conservation Center for Wild Endangered Plant Resources, Shanghai Chenshan Botanical Garden, Shanghai 201602, China Shanghai Chenshan Botanical Garden Shanghai China

**Keywords:** cpDNA, Lamiales, molecular phylogenetics, morphology, nrITS

## Abstract

*Mazusdanxiacola*, a new species endemic to Danxia landform in east Jiangxi Province, eastern China, is described and illustrated. The systematic placement of this new species was confirmed by molecular phylogenetic analyses based on four plastid markers (*matK*, *rbcL*, *rps16* and *trnL-trnF*) and nuclear ribosome ITS sequence, and its specific relationships within *Mazus* were discussed. Morphologically, the new species is clearly different from other *Mazus* species by having a series of uncommon traits, i.e., annual habit, without stolons and basal leaves, single, erect and unbranched stems, long petiolate leaves abaxially grayish green to silver gray, truncate to broadly cuneate leaf bases, racemes extremely elongated up to 35 cm long, white corolla, and palate densely covered by conspicuous clavate gland-like hairs. The new species is assigned to Critically Endangered (CR) according to the IUCN Red List Categories and Criteria.

## ﻿Introduction

Mazaceae (Reveal 2011) is a newly established small family that was separated from the traditionally circumscribed Scrophulariaceae (e.g., Von Wettstein 1891; Thieret 1967; Fischer 2004). There are four genera currently recognized in the family: *Dodartia* L., *Lancea* Hook.f. & Thomson, *Mazus* Lour, and *Puchiumazus* Bo Li, D.G. Zhang & C.L. Xiang (Stevens 2001 onwards; Xiang et al. 2021). Among these, *Dodartia*, *Lancea* and *Puchiumazus* contains only sole or two species (Fischer 2004; Deng et al. 2019; Xiang et al. 2021), while *Mazus* includes 37 accepted species which are distributed mainly in Asia to Australasia (POWO 2022). Nearly all species of *Mazus* are annual or perennial herbs (Hong et al. 1998; Deng et al. 2016), except the *M.fruticosus* Bo Li, D.G. Zhang & C.L. Xiang which was recently described as a new species having a shrubby habit (Xiang et al. 2021). *Mazus* is characterized by a combination of morphological characters: a strongly two-lipped corolla (3/2-bilabiatae), a palate with two longitudinal plaits and a capsule enclosed in a persistent calyx (Fischer 2004; Deng et al. 2019). In China, 25 species and three varieties were recorded in the *Flora of China* (*FOC*, Hong et al. 1998), but new species were continuously reported since the publication of the *FOC*, i.e., *M.tainanensis* T.H. Hsieh (Hsieh 2000), *M.sunhangii* D.G. Zhang & T. Deng (Deng et al. 2016), *M.somggangensis* S.S. Ying (Ying 2019), *M.fruticosus* (Xiang et al. 2021), etc., indicating that there is probably a hidden diversity of *Mazus* in China that needs to be revealed.

**Figure 1.**
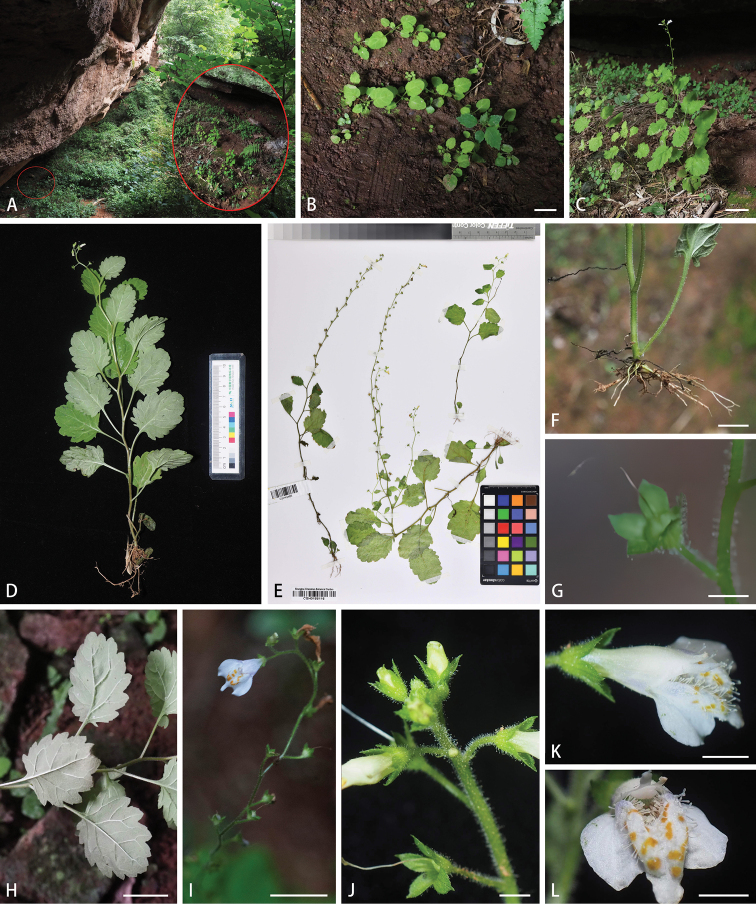
Morphology of *Mazusdanxiacola* sp. nov. **A** habitat **B** seedlings **C** habit **D** a flowering individual **E** fruiting specimens **F** roots **G** fruit **H** leaves **I** a mature inflorescence with flowers and fruits **J** young inflorescence, showing dense pubescence and glandular hairs **K** flower (lateral view) **L** palate, showing the conspicuously long and clavate gland-like hairs. Scale bars: 2 cm (**B, H**); 5 cm (**C**); 1 cm (**F, I**); 2 mm (**G, J, K, L**).

In 2021, during a special botanical survey for the Danxia landforms in Jiangxi Province, eastern China, the authors encountered two populations of an unusual species of *Mazus* in Guixi City, eastern Jiangxi. The unknown plant is an annual herb having a single erect unbranched stem, no rosulate basal leaves, stem leaves many and alternate with long petioles up to 4.5 cm, abaxial leaf surface grayish green to silver gray, raceme extremely elongated up to 35 cm and densely pubescent and glandular hairs, white corolla with the palate densely covering conspicuous and clavate gland-like hairs (Fig. 1). After checking and comparing the plant with all known congeneric taxa, we conclude that it represents a distinct undescribed new species of *Mazus*, *M.danxiacola*, which is described in the present study.

## ﻿Materials and methods

Field investigations were carried out in Danxia mountains of Guixi City, Jiangxi Province from May to October in 2021. Voucher specimens in flowering and fruiting were collected in the field in June and August, respectively. All specimens were deposited in the herbarium of Shanghai Chenshan Botanical Garden (CSH) and voucher photos taken *in situ* were deposited in the “Chinese Field Herbarium” (https://www.cfh.ac.cn/album/ShowSpAlbum.aspx?spid=94285).

The morphological description of the putative new species was conducted based on observations and measurements of mature plants in field as well as specimens. Measurements were taken using a ruler and a vernier caliper. Herbarium specimens of other *Mazus* species in China were examined via the Chinese Virtual Herbarium (https://www.cvh.ac.cn/) and National Specimen Information Infrastructure (http://www.nsii.org.cn/2017/home.php) platforms. High resolution images of the type specimens of *Mazus* were consulted on JSTOR Global Plants (http://about.jstor.org/). The conservation status of the new species was evaluated based on the guidelines of the IUCN Red List categories and criteria (IUCN 2022).

In order to confirm the systematic placement of the putative new species within *Mazus*, molecular phylogenetic analyses were conducted following the procedures presented in Xiang et al. (2021). The combined cpDNA dataset (*matK*, *rbcL*, *rps16* and *trnL*-*trnF*) and the nrITS dataset used in Xiang et al. (2021) were employed with the addition of two individuals (*B.Chen CB06425* and *B.Chen CB05735*) of the putative new species. The two datasets were simplified and adjusted to set the species of *Mazus* as ingroups (22 and 17 species in cpDNA and nrITS datasets, respectively) and *Dodartiaorientalis* L. and *Lanceatibetica* Hook. f. & Thomson were selected as outgroups based on previous phylogenies (Deng et al. 2019; Xiang et al. 2021). Methods of DNA extraction, amplification, sequencing, and phylogenetic analyses using maximum likelihood (ML) and Bayesian inference (BI) follow those presented in Deng et al. (2019) and Xiang et al. (2021). Voucher information and GenBank accession numbers for taxa used in this study are provided in Table 1.

**Table 1.** Taxa, GenBank accession numbers of DNA sequences, and their vouchers used in this study. Newly sequenced taxa are shown in bold, and missing data are indicated by a dash (-).

Taxa	matK	Voucher	rbcL	voucher	rps16	voucher	trnL-F	voucher	ITS	voucher
*Dodartiaorientalis* L.	MK392230	*XZ-2008-1*	JQ342984	*XZ-2008-1*	JQ342982	*XZ-2008-1*	JQ342981	*XZ-2008-1*	JQ342980	*XZ-2008-1*
*Lanceatibetica* Hook. f. & Thomson	MF786907	*Tibet-MacArthur2276*	MF786661	*Tibet-MacArthur2276*	FJ172699	*XZ-2007-0525*	FJ172685	*XZ-2007-0525*	FJ172736	*XZ-2007-0525*
*Mazusalpinus* Masam.	MK266256	*Sunhang11307*	KX783481	*Sunhang11307*	KX783501	*Sunhang11307*	KX783520	*Sunhang11307*	MK192641	*Sunhang11307*
*M.caducifer* Hance	MK266277	*KUN35025*	KX783477	*KUN35025*	KX783497	*KUN35025*	KX783516	*KUN35025*	MK192664	*KUN35025*
*M.celsioides* Hand.-Mazz.	–	–	KX783486	*YIF0093*	MK266366	*YIF0093*	KX783525	*YIF0093*	–	–
***M.danxiacola* Bo Li & B. Chen 1**	**ON323563**	***CB06425***	**ON323565**	***CB06425***	**ON323567**	***CB06425***	**ON323569**	***CB06425***	**ON286711**	***CB06425***
***M.danxiacola* Bo Li & B. Chen 2**	**ON323564**	***CB05735***	**ON323566**	***CB05735***	**ON323568**	***CB05735***	**ON323570**	***CB05735***	**ON303604**	***CB05735***
*M.fauriei* Bonati	–	–	KX783479	*Sunhang11248*	KX783499	*Sunhang11248*	KX783518	*Sunhang11248*	LC034207	*HUP97*
*M.gracilis* Hemsl.	–	–	FJ172729	*XZ-2007-058*	FJ172701	*XZ-2007-058*	FJ172687	*XZ-2007-058*	FJ172738	*XZ-2007-058*
*M.fruticosus* Bo Li, D.G. Zhang & C.L. Xiang	MK266261	*zdg4447*	KX783470	*zdg4447*	KX783490	*zdg4447*	KX783509	*zdg4447*	MK192660	*zdg4447*
*M.humilis* Hand.-Mazz.	–	–	–	–	MK266367	*dt149*	MK266421	*dt149*	MK192667	*dt149*
*M.longipes* Bonati	MK266267	*Deng1941*	KX783474	*Deng1941*	KX783494	*Deng1941*	KX783513	*Deng1941*	MK192652	*Deng1941*
*M.miquelii* Makino	NC_056339	*Zeng et al. (2021)*	NC_056339	*Zeng et al. (2021)*	NC_056339	*Zeng et al. (2021)*	NC_056339	*Zeng et al. (2021)*	LC027734	*Maruyama:sn*
*M.novaezeelandiae* W.R. Barker	MK266278	*dtA68*	KX783469	*dtA68*	KX783489	*dtA68*	KX783508	*dtA68*	MK192676	*dtA68*
*M.omeiensis* H.L. Li	MK266252	*nie1976*	KX807209	*nie1976*	KX807203	*nie1976*	KX807208	*nie1976*	MK192636	*nie1976*
*M.procumbens* Hemsl.	MK266261	*zdg6074*	KX783478	*zdg6074*	KX783498	*zdg6074*	KX783517	*zdg6074*	MK192647	*zdg6074*
*M.pulchellus* Hemsl.	–	–	KX783472	*dt093*	KX783492	*dt093*	KX783511	*dt093*	MK192638	*dt093*
*M.pumilio* R. Br.	MK266277	*Pagest.s.n.2021829*	KX783468	*Pagest.s.n.2021829*	KX783488	*Pagest.s.n.2021829*	KX783507	*Pagest.s.n.2021829*	MK192671	*Pagest.s.n.2021829*
*M.pumilus* (Burm. f.) Steenis	MK266259	*XZ-2007-051*	FJ172728	*XZ-2007-051*	FJ172700	*XZ-2007-051*	FJ172686	*XZ-2007-051*	FJ172737	*XZ-2007-051*
*M.pumilus var. delavayi* (Bonati) T.L. Chin ex D.Y. Hong	MK266257	*Sunhang11459*	KX783482	*Sunhang11459*	KX783502	*Sunhang11459*	KX783521	*Sunhang11459*	–	–
*M.radicans* Cheesman	–	–	KT626738	*CHR618785*	MK266381	*CHR618785*	–	–	MK192635	*CHR618785*
*M.spicatus* Vaniot	MK266251	*XZ-2007-0514*	FJ172730	*XZ-2007-0514*	FJ172703	*XZ-2007-0514*	FJ172689	*XZ-2007-0514*	FJ172740	*XZ-2007-0514*
*M.sunhangii* D.G. Zhang & T. Deng	–	–	KX783484	*zdg4142*	KX783504	*zdg4142*	KX783523	*zdg4142*	–	–
*M.surculosus* D. Don	–	–	KX783473	*KUN0472212*	KX783493	*KUN0472212*	KX783512	*KUN0472212*	–	–
*M.xiuningensis* X.H. Guo & X.L. Liu	NC_056340	*Zeng et al. (2021)*	NC_056340	*Zeng et al. (2021)*	NC_056340	*Zeng et al. (2021)*	NC_056340	*Zeng et al. (2021)*	–	–

## ﻿Results

### ﻿Phylogenetic analysis

**Figure 2.**
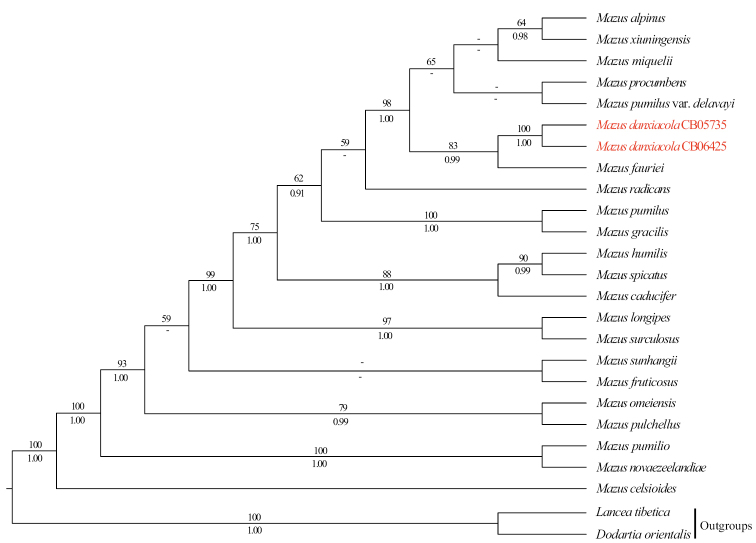
Maximum Likelihood phylogram of *Mazus* as inferred from analysis of combined dataset of *matK*, *rbcL*, *rps16* and *trnL*-*trnF*. Support values ≥ 50% BS or 0.90 PP are displayed above and below the branches, respectively.

**Figure 3.**
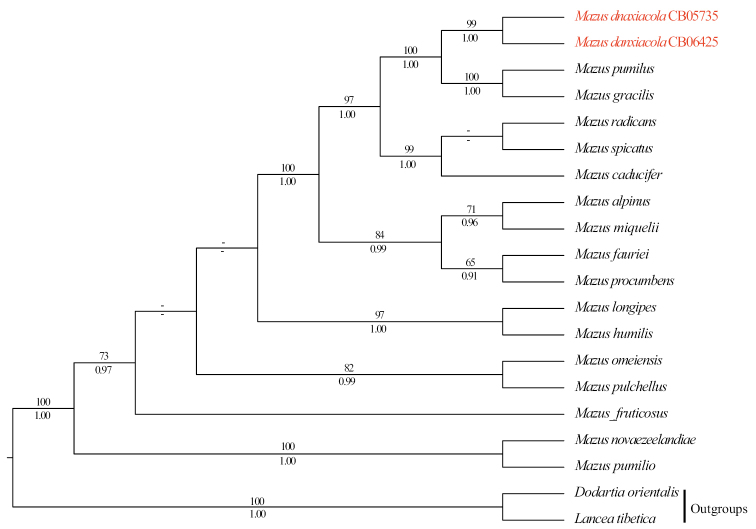
Maximum Likelihood phylogram of *Mazus* as inferred from analysis of nrITS dataset. Support values ≥ 50% BS or 0.90 PP are displayed above and below the branches, respectively.

The combined cpDNA dataset has 25 aligned sequences and comprise 3851 characters (860 bp for *matK*, 1267 bp for *rbcL*, 837 bp for *rps16*, and 887bp for *trnL-trnF*, respectively), of which 327 are variable (8.49%) and 225 are parsimony-informative (5.84%). The nrITS dataset has 20 sequences with the aligned length of 609 bp, of which 176 are variable (28.90%) and 142 are parsimony-informative (23.32%). Phylogenetic analyses based on the two datasets were conducted separately because the taxon sampling is different in these datasets. ML and BI trees generated from each dataset yielded similar topologies, thus only the ML trees are presented (Figs 2, 3). In all analyses, the monophyly of *Mazus* was strongly supported (Figs 2, 3; cpDNA tree: ML-BS=100%, BI-PP=1.00; nrITS tree: ML-BS=100%, BI-PP=1.00; all values reported in this order below), and the two individuals of *M.danxiacola* formed a highly supported clade (99%, 1.00; 99%, 0.99), which was consistently nested within *Mazus* in both cpDNA and nrITS trees. However, specific relationships within the genus were not fully resolved. In the cpDNA tree, *M.danxiacola* was sister to *M.fauriei* Bonati with moderate supports (Fig. 2; 83%, 0.99), while in the nrITS tree, *M.danxiacola* was sister to a clade comprising *M.pumilis* (N.L. Burman) Steenis and *M.gracilis* Hemsl. (100%, 1.00), and they together obtained highly supported values (100%, 1.00).

### ﻿Taxonomic treatment

#### ﻿ Mazus danxiacola

Taxon classificationPlantaeLamialesMazaceae

Bo Li & B. Chen sp. nov.

5DED7DFE-4570-53C3-A1AC-23B14642B08E

urn:lsid:ipni.org:names:77299061-1

Fig. 1

##### Diagnosis.

This species is distinct from all currently known congeneric species and could be easily distinguishable by its annual habit, single, erect and unbranched stems, long petiolate leaves with truncate to broadly cuneate base and grayish green to silver gray lower surface, terminal racemes up to 35 cm long, white corolla with the palate densely covering conspicuous clavate gland-like hairs and having no stolons and basal leaves.

##### Type.

China. Jiangxi Province: Guixi City, Liukou town, under the cliffs of Danxia mountains, alt. 75m a.s.l., 12 June 2021, *Bin Chen CB05735* (holotype CSH!, barcode CSH0186434; isotypes CSH!, barcode CSH0186431, CSH0186433, CSH0118470); in the same location of holotype, 24 August 2021, *Xingui Le & Lin Xu CSH42465* (paratype CSH!, barcode CSH0188116).

##### Description.

Annual herbs, 15–65 cm tall, without stolons. Primary roots thick and strong; adventitious roots numerous, shotting from the stem base, white and slightly freshy. Stems single, erect, unbranched, terete; old stems purplish brown, sparsely puberulent; young stems grayish green, densely villous and sparsely glandular hairy. Leaves all cauline, numerous, alternate, long petiolate, larger at middle of stem; petioles densely puberulent to subglabrous, 1.5–4.5 cm long; leaf blade broadly ovate to suborbicular, membranous, 2.5–5.3 × 2.3–4.8 cm, adaxially green, subglabrous to sparsely puberulent, abaxially grayish green to silver gray, subglabrous, densely villous on veins, apex obtuse to rounded, base truncate to broadly cuneate, margin crenate, teeth apices callous, sometimes with 1 or 2 pairs of lobes near base; veins conspicuous on both surface, elevated abaxially, fluted adaxially. Racemes terminal or occasionally axillary on the top 1–3 nodes, shortened when young but elongated up to 35 cm long when fruiting, lax, multiflowered; pedicels slender, 0.8–2.5 cm long, densely villous and glandular hairy. Calyces broadly campanulate, 3.0–4.0 mm long, 5-veined, densely villous and glandular hairy outside, subglabrous inside; lobes 5, ovate-triangular, longer than the tube, apex acute, midrib conspicuous, lateral veins inconspicuous. Corolla white, dotted yellow on palate, 0.9–1.2 cm long, sparsely minutely puberulent to glabrous outside, tube cylindric, 0.4–0.6 cm long, exserted from calyx; limb 2-lipped, upper lip bilobed, upwarp, lobes lanceolate; lower lip trilobed, middle lobe narrowly ovate, ca. 1.5 mm long, smaller than lateral lobes, lateral lobes spreading away from middle lobes, broadly ovate to rectangular; palate comprising 2 longitudinal elevations extending from point of filament fusion to base of lower lobes, densely covered by gland-like hairs, hairs clavate and conspicuous, ca. 0.7 mm long, white to transparent. Stamens 4, didynamous, glabrous, inserted at the same level in distal part of tube, included; anterior pair longer, curved, appressed to corolla tube, posterior pair spreading; anthers bithecal, positioned adjacent to corolla tube on upper lip; filaments filiform, glabrous. Styles 0.4–0.5 cm long, included, exserted beyond anthers, stigma 2-lamellate. Capsule globose, ca. 2.5 mm in diam, apex rounded, included by persistent calyx.

##### Phenology.

Flowering was observed from early June to late August and fruiting from June to late September.

##### Distribution and habitat.

**Figure 4.**
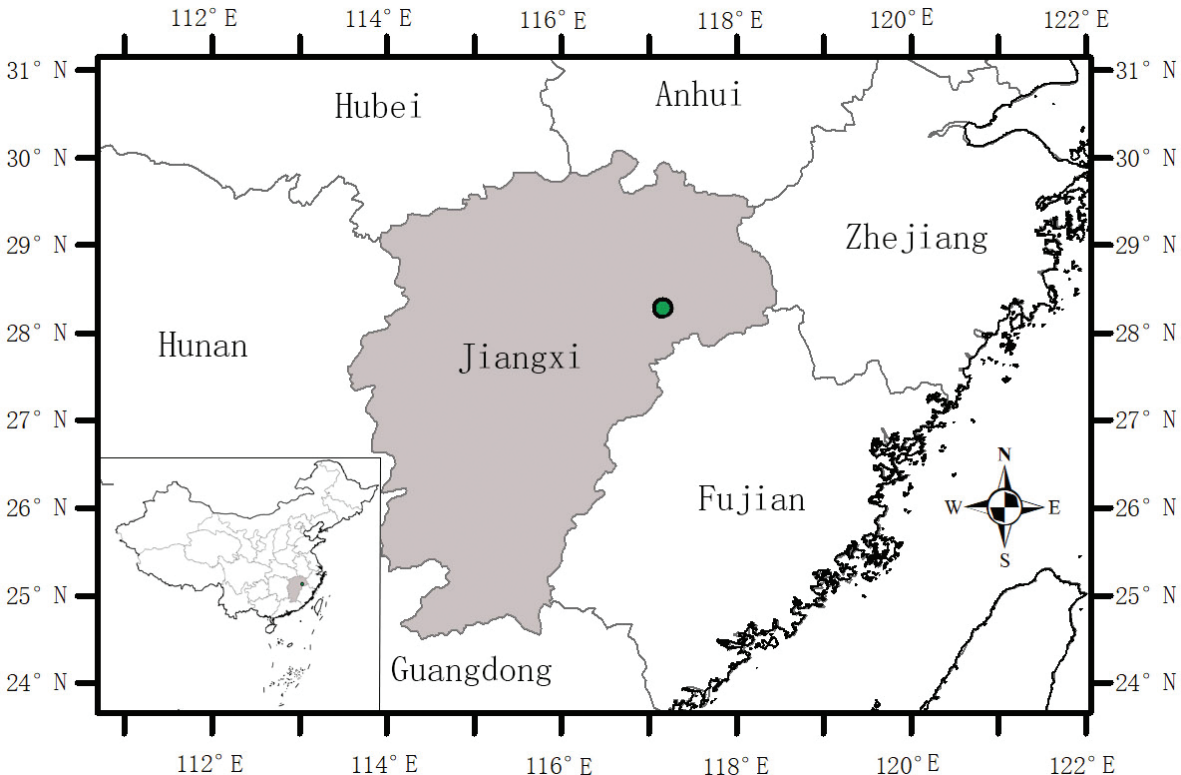
Distribution map of *Mazusdanxiacola* sp. nov.

**Figure 5.**
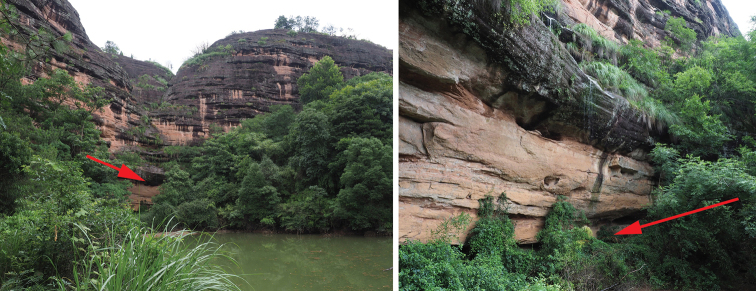
Danxia landform of the type locality of *Mazusdanxiacola* sp. nov. (left) and the habitat of the new species under a cliff (right). Arrows show where the population could be found.

The species is currently known only from the type locality of Danxia mountains in Liukou Town of Guixi City, eastern Jiangxi Province, China (Fig. 4), and grows under shaded cliffs and near the edges of subtropical evergreen broad-leaved forests, at an elevation about 75 m a.s.l. (Figs 1A, 5).

##### Etymology.

The specific epithet “*danxiacola*” refers to the species inhabiting in Danxia landform.

##### Vernacular name.

Simplified Chinese: 丹霞通泉草; Chinese pinyin: Dān Xiá Tōng Quán Căo.

##### Provisional conservation status.

Based on our special botanical surveys for Danxia landforms in Jiangxi Province in 2021, *M.danxiacola* has been discovered only from one single locality so far in Liukou Town of Guixi City in Jiangxi Province, China, and 2 populations were found in the locality, which totally occupied ca. 200 m^2^. In these populations, a total of ca. 80 fruiting individuals were counted in August 2021 and there were a lot of seedlings in each of the population when we firstly encountered the species in June 2021 (Fig. 1B, C), indicating that the species has a well-developed reproductive strategy in the habitat of Danxia landform. However, the locality is close to downtown of Guixi City, has not been projected to a nature reserve yet and all populations are obviously facing man-made interferences, such as deforestation, touring and grazing, we thus propose to categorize the species as critically endangered (CR) under criteria B and D following IUCN Red List Categories (IUCN 2022).

##### Taxonomic note.

Morphologically, *M.danxiacola* bears a series of rare traits which are not common in *Mazus*, such as annual habit, single erect unbranched stems, without basal leaves, stem leaves many and alternate, extremely long petioles up to 4.5 cm, abaxial leaf surface grayish green to silver gray, and palate of corolla densely covered by conspicuously clavate gland-like hairs. The combination of these traits makes *M.danxiacola* distinct from all other congeneric taxa. Our molecular phylogenetic analyses based on cpDNA dataset indicated that *M.fauriei* may be closely related to *M.danxiacola* (Fig. 2), but *M.fauriei* is a perennial herb with all leaves basal and rosulate and petioles broadly winged (Hong et al. 1998), which is apparently different from *M.danxiacola* that has only cauline leaves and long unwinged petioles. In the nrITS trees, *M.pumilis* and *M.gracilis* were shown as possible alliances of *M.danxiacola*, however, cauline leaves of the former two species are always opposite, and their basal and cauline leaves are all decurrent to form short petioles (Hong et al. 1998), clearly differing from those alternate and long petiolate leaves of *M.danxiacola*. It is worth mentioning that there are obvious conflicts between the cpDNA and nrITS phylogenies which have been discovered and discussed in a previous study (Xiang et al. 2021). In fact, the available molecular data of *Mazus* were not sufficient enough to represent all known species of the genus, thus it is hard to definitely confirm the closest relatives of *M.danxiacola* at the moment through molecular phylogenetics. Future molecular studies including more species at population level and using more DNA markers may shed light on the determination of specific relationships within *Mazus*.

So far, *M.danxiacola* is the first species of *Mazus* that was found to be endemic to Danxia landform. Danxia landform is a unique type of petrographic geomorphology found in southeast, southwest, and northwest China with a high level of floral endemism (Liu et al. 1999; Liu and Liu 2003; Luo et al. 2010). In southeast China, Danxia landforms are well developed in Guangdong, Fujian, Jiangxi, and Hunan provinces, and the special environment, including deep valleys, grooves, moist caves, cliffy rocks, dry cliff-tops and shaded rock bottoms (Fig. 5), has significant effects on the growth of special plants (Chen et al. 2008). Just in the last ten years, a lot of new taxa have been continuously discovered from Danxia mountains of these provinces, i.e., *Danxiaorchis* J.W. Zhai, F.W. Xing & Z.J. Liu (Zhai et al. 2013), *Spiradiclisdanxiashanensis* R.J. Wang (Wang et al. 2015), *Violahybanthoides* W.B. Liao & Q. Fan (Fan et al. 2015), *Begoniadanxiaensis* D.K. Tian & X.L. Yu (Tian et al. 2019), *Phyllostachysdanxiashanensis* N.H. Xia & X.R. Zheng (Zheng et al. 2019), *Semiaquilegiadanxiashanensis* L. Wu, J.J. Zhou, Qiang Zhang & W.S. Deng (Zhou et al. 2019), *Lespedezadanxiaensis* Q. Fan, W.Y. Zhao & K.W. Jiang (Zhao et al. 2021), *Aspleniumdanxiaense* K.W. Xu et al. (2022), etc., indicating that it is valuable to strengthen the flora investigations in Danxia landforms and uncover the biodiversity.

## Supplementary Material

XML Treatment for Mazus danxiacola
